# Extracorporeal Membrane Oxygenation for Septic Shock in Adults and Children: A Narrative Review

**DOI:** 10.3390/jcm12206661

**Published:** 2023-10-20

**Authors:** Lars Mikael Broman, Olga Dubrovskaja, Martin Balik

**Affiliations:** 1 ECMO Centre Karolinska, Pediatric Perioperative Medicine and Intensive Care, Karolinska University Hospital, 17176 Stockholm, Sweden; 2 Department of Physiology and Pharmacology, Karolinska Institutet, 17177 Stockholm, Sweden; 3 Intensive Care Department II, North Estonia Medical Centre, 13419 Tallinn, Estonia; olga.dubrovskaja@regionaalhaigla.ee; 4 Department of Anesthesiology and Intensive Care, 1st Faculty of Medicine, Charles University and General University Hospital in Prague, 12808 Prague, Czech Republic; martin.balik@vfn.cz

**Keywords:** extracorporeal membrane oxygenation, septic shock, refractory vasoparalysis, cardiac output, stress cardiomyopathy, distributive shock

## Abstract

Refractory septic shock is associated with a high risk of death. Circulatory support in the form of veno-arterial extracorporeal membrane oxygenation (VA ECMO) may function as a bridge to recovery, allowing for the treatment of the source of the sepsis. Whilst VA ECMO has been accepted as the means of hemodynamic support for children, in adults, single center observational studies show survival rates of only 70–90% for hypodynamic septic shock. The use of VA ECMO for circulatory support in hyperdynamic septic shock with preserved cardiac output or when applied late during cardio-pulmonary resuscitation is not recommended. With unresolving septic shock and a loss of ventriculo–arterial coupling, stress cardiomyopathy often develops. If the cardiac index (CI) approaches subnormal levels (CI < 2.5 L/min m^−2^) that do not match low systemic vascular resistance with a resulting loss of vital systemic perfusion pressure, VA ECMO support should be considered. A further decrease to the level of cardiogenic shock (CI < 1.8 L/min m^−2^) should be regarded as an indication for VA ECMO insertion. For patients who maintain a normal-to-high CI as part of their refractory vasoparalysis, VA ECMO support is justified in children and possibly in patients with a low body mass index. Extracorporeal support for septic shock should be limited to high-volume ECMO centers.

## 1. Introduction

Sepsis is a worldwide healthcare problem caused by the dysregulation of the host’s response to infection and was recently estimated to cause 20% of all global deaths, represented by approximately 11 million cases in 2017 [1,2]. In the United States, the incidence of sepsis is twice that of acute myocardial infarction (AMI) (https://nigms.nih.gov/education/fact-sheets/Pages/sepsis.aspx (accessed on 7 October 2023)), and accounts for more deaths than AMI in adults (https://www.cdc.gov/heartdisease/facts.htm (accessed on 7 October 2023)) [3]. Every third hospital death is due to sepsis, whereas cardiac disease, in general, stands for about 20%. Likewise, septic shock is known to carry among the highest risks of death in intensive care [1,2,4], as well as a significant burden of morbidity and long-term suffering among survivors [5,6,7]. The incidence of severe sepsis in the pediatric population is approximately 0.6–0.9/1.000 and the mortality ranges between 10 and 25%, with a certain risk of disability [5,6,7]. Extracorporeal membrane oxygenation (ECMO) has been used for a few decades and is accepted as the last resort support for neonatal and pediatric patients with septic shock [8,9,10,11,12]. In these children, the mortality is generally above 50%, with the exception of a limited number of single center reports [13,14,15,16,17,18].

Concerning newborns, sepsis is reported to be the second most common cause of death in preterm infants [19], and the most common cause of death in middle- and low-income countries [20]. Early onset sepsis, within 72 h from birth, is regarded as genitourinary transmission from the mother to the fetus or the newborn. Late onset sepsis begins >72 h from birth and is due to the transmission of microorganisms from the surrounding environment [21,22].

In adults, the utilization of ECMO in refractory septic shock shows increasing numbers; nevertheless, it is still not considered a standard treatment [9,21]. Despite causing discussions concerning its usefulness [23,24], several recent publications support the use of ECMO for sepsis in adults [17,18,25,26,27]. Single center studies, with their known inherent limitations, show survival rates of 70–90% in hypodynamic (i.e., septic cardiomyopathy) septic shock, with estimated mortality rates above 60%, according to contemporary risk stratification models [17,18,28]. However, the available data do not support the use of ECMO in adults or adolescents with hyperdynamic (distributive) sepsis and preserved cardiac output in, for example, meningococcal septic shock [29], especially in combination with extracorporeal cardio-pulmonary resuscitation [26,27]. 

This narrative review summarizes the current state-of-the-art ECMO support in septic shock and attempts to offer a practical approach to its indication at the bedside. 

## 2. General

### 2.1. Current Position of ECMO in Guidelines for Septic Shock Management 

In addition to the Sepsis-3 definition for adults [2], pediatrics [30], and neonates for sepsis [22], international societies and other stakeholders have also published guidelines. The criteria for sepsis in children still rely on systemic inflammatory response syndrome (SIRS), suspected or confirmed infection, and a level of cardiovascular impairment [31,32]. In the latest revision of the Surviving Sepsis Campaign (2021) [33,34], veno-venous (VV) ECMO is recommended in respiratory failure (low degree of evidence). No recommendation is published on sepsis-associated cardiac failure. Regarding pediatric sepsis, the American College of Critical Care Medicine and the Society of Critical Care Medicine published the latest revised guidelines in 2017, recommending ECMO as a last resort therapy in refractory septic shock [35,36]. Kawasaki et al. underlined that this recommendation only relied on three publications [6,14,15,16]. International guidelines on ECMO specifically are revised and published regularly by the Extracorporeal Life Support Organization (ELSO, Ann Arbor, MI, USA) [37,38,39,40], and are available online (https://www.elso.org/ecmo-resources/elso-ecmo-guidelines.aspx (accessed on 7 October 2023)).

### 2.2. Terminology and Nomenclature

Due to a lack of alignment in terminology and communication in the expanding field of extracorporeal life support (ECLS), in 2018 and 2019, the ELSO published two position papers on terminology, nomenclature, and cannulation abbreviations in the ECLS [41,42]. In short, in addition to the recently acknowledged veno-pulmonary (VP) [43], veno-venous (VV), veno-arterial (VA), and the hybrid veno-venoarterial (VVA) and veno-pulmoarterial (VPA), there are the five modalities (modes) of ECLS. The next abbreviation step formulates the cannulation configuration. The abbreviation reads from left to right, starting with the drainage/drainage cannula, followed by a hyphen that marks the membrane lung (ML, oxygenator). The position to the right of the hyphen is the return. To exemplify, V-V denotes the VV ECMO mode with one venous drainage cannula, one ML, and one return cannula placed in a vein. The next step continues with which vessels that are cannulated, etc. [42]. The abbreviation formulation is hierarchical and offers several layers of depth, and all combinations of ECLS can be described. In this work, we will not go into more detail than necessary. 

### 2.3. Indications

The indications for ECMO in septic shock include both respiratory and circulatory criteria and are often a combination of the failure of both organ systems. The decision should be made on an individual level based on knowledge of the patient’s disease, patient age, institutional experience, expert consensus, and consultation. Support may be found in the ELSO Guidelines [37,38,39,40], (https://www.elso.org/ecmo-resources/elso-ecmo-guidelines.aspx (accessed on 7 October 2023)). An age- and organ-related summary is provided in Table 1. 

### 2.4. Use of ECMO for Septic Shock in Different Age Groups

#### 2.4.1. Neonate (0–28 Days of Age)

Early onset sepsis (i.e., within 72 h from birth) is commonly caused by the genitourinary transmission of bacterial infections [21,22]. The maternal risk factors are GBS colonization, chorioamnionitis, delivery before gestational week 37, and ruptured amniotic sack >18 h. Late onset (>72 h from birth) generally occurs through the transmission of microorganisms from the environment; its risk factors are indwelling catheters, other invasive procedures, etc. In a recent review on neonatal and pediatric sepsis, the overall survival from hospital was 59% [45]. The ECMO duration was 5.4 days, the pooled survival from VA ECMO was 65%, and the survival rate in neonates was 73%. The authors concluded that ECMO should be considered for refractory septic shock in all pediatric age groups. The data also support the transfer of these patients to ECMO referral centers [46]. 

#### 2.4.2. Pediatric (1 Month-18 Years)

Studies on sepsis in children are few and patient numbers are limited. Reports from the 1990s (including ELSO Registry data) indicated survival rates of between 36 and >50%, and it was concluded that ECMO should not be withheld from children with septic shock [47,48]. The two pivotal studies from MacLaren et al. (2007 and 2011), with survival rates of 73–74%, led to the conclusion that the septic child may benefit from central cannulation veno-arterial (VA) ECMO, allowing for higher ECMO blood flows compared to typical peripheral cannulation [14,15]. Many centers have adopted this concept; however, in a recent retrospective single-center study on peripheral cannulation, the survival was comparable to central cannulation in similarly sick patients. In this work, the VA ECMO blood flow was 80–100 mL/kg min^−1^ [13], thus not aiming for hyper-perfusion (150–400 mL/kg min^−1^), as suggested earlier [49,50,51].

#### 2.4.3. Adult Population (>18 Years)

Bréchot and co-workers reported a 71% survival rate and low post-ECMO mortality in patients with cardiac failure on top of severe septic shock, e.g., hypodynamic septic shock [18], and similar results were also reported by others [17,52]. However, the combination of sepsis and ECMO-assisted cardiopulmonary resuscitation is dismal [26,27]. A meta-analysis confirmed the physiologic rationale for VA ECMO as circulatory support in septic shock with associated ventricular dysfunction [53]. A summary of the clinically relevant publications is provided in Supplementary Table S1.

### 2.5. Physiology and Cannulation Strategies

To understand the oxygenation and extracorporeal blood flow distribution profile of the patient’s circulation after ECMO implantation, knowledge of the cannula design and positioning is crucial. The drainage cannula not only limits the drainage—i.e., ECMO blood flow—but also the overall oxygen distribution in the patient [54,55,56,57,58]. The aim is to apply the largest caliber drainage (venous) cannula to allow for drainage at a low applied suction pressure (≥−80 mmHg). In the decision-making process, ultrasound/echocardiography is an important tool for assessing both the cardiac function and the vessel size(s) before cannulation. Moreover, a detailed drainage profile of the drainage zone of, in particular, the multi-staged or bi-caval single lumen cannula may impact the patient’s stability during, for example, changing preload [59,60,61]. Table S2 shows different cannulation strategies.

**Figure 1:**
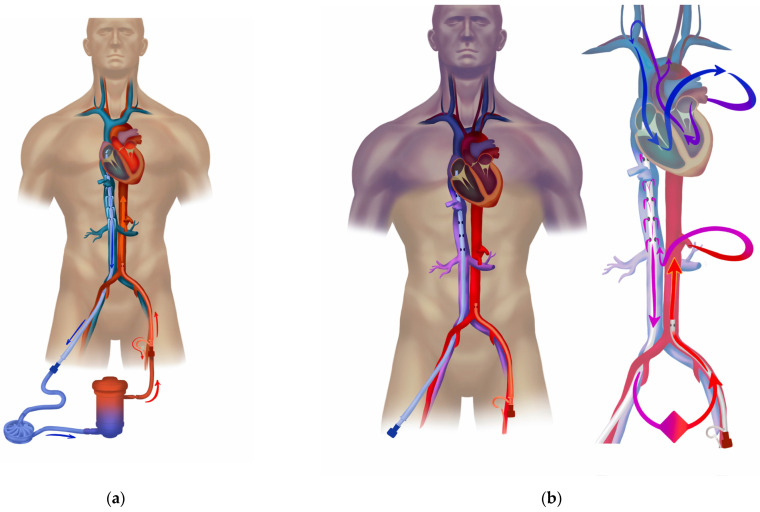
Femoro-femoral veno-arterial extracorporeal membrane oxygenation. Panel (**a**): Typical femoro-femoral veno-arterial (VA) extracorporeal membrane oxygenation (ECMO) with drainage cannula tip in right atrium (RA) and return into the lower abdominal aorta (or iliac artery). The cannulated leg is perfused with a distal perfusion catheter. Drainage cannula placement with tip in upper of right atrium or into the lower part of the superior vena cava (SVC) provides efficient drainage of low saturated venous blood. Use of a single stage (lighthouse tip) limits the risk of severe differential oxygenation in case of worsening of lung function. A patient with septic shock due to pneumonia (**b**): A multi-stage cannula placed via a femoral vein, drains most of the flow via the most proximal side-holes and these are positioned in the inferior vena cava (drainage zone below the diaphragm). Thus, the risk of fulminant differential hypoxemia in the upper body is higher with such configuration when the heart recovers but the lungs are still not working (Panel (**b**)). ELSO configuration abbreviation: V_fra_-A_fl_d_t_.

The pathophysiology of distributive shock due to sepsis lies in altered systemic vascular resistance (SVR), which leads to hypotension with a peripheral imbalance between oxygen delivery and consumption (DO_2_/VO_2_). A low SVR is, at least initially, compensated for by an increased cardiac output, maintaining vital perfusion pressure. With a gradual loss of coupling between heart elastance (especially left ventricular contractility) and arteriolar elastance (SVR), the heart compensation may not be sufficient [62]. Typically, during 48–72 h of unresolving septic shock, cardiomyopathy develops [63,64], presenting as *stress,* or Takotsubo cardiomyopathy [65]. In cases of inadequate cardiac output not matching a low SVR, hypodynamic septic shock develops, where a circulatory support with an extra blood flow of VA ECMO may establish vital perfusion pressures and peripheral oxygen delivery (Figure 1) [17,18,52,66]. Here, VA ECMO is launched as a bridge to recovery, also allowing for at least partial weaning of catecholamines [67] and a limitation of their adverse impact on stressed cardiomyocytes [68,69,70,71]. Typically, the patient presents with a complicated management of the source of sepsis (e.g., pneumonia due to resistant bacteria) and profound refractory vasoparalysis. After the VA ECMO insertion, repeated echocardiographic assessment is warranted, enabling titration of the ECMO blood flow and patient preload to limit the negative impact of the increased cardiac afterload caused by VA ECMO. In addition to cardiac protection and the reduction in catecholamines, the maintenance of minimum cardiac output to secure heart unloading is crucial for the outcome of the VA ECMO therapy. If successful, heart recovery and a gradual increase in cardiac output lead to hypodynamic septic shock turning into hyperdynamic with persisting low systemic vascular resistance [52,72]. Hence, anticipating the requirement for higher ECMO blood flow is important, which is also part of the pediatric recommendations [14,15]. A requirement for high flows, circulating stress volume, and the management of a hyperkinetic stage of septic shock are dependent on the patient’s body mass index and body weight. Inevitably, the higher ECMO blood flows in septic shock must involve large diameter cannulas (e.g., drainage 25–29 F, return 21–23 F) and prior vessel assessment through ultrasound in cases of peripheral VA ECMO. A large-diameter return cannula implicates a routine prograde cannulation of the femoral superficial artery of the cannulated leg to secure the distal leg perfusion (Figure 1). This approach also has an implication for the later explanation of the VA ECMO, which requires surgical extraction through a thrombectomy from the low-flow segment between the return cannula and the leg perfusion catheter. Some centers apply a distal leg perfusion, where a thinner arterial line is introduced in an artery at the ankle. VA ECMO allows for the lung-protective spontaneously triggered or spontaneous ventilation of the oligemic lungs as most of the right heart blood volume is initially unloaded into the extracorporeal circuit. 

**Figure 2:**
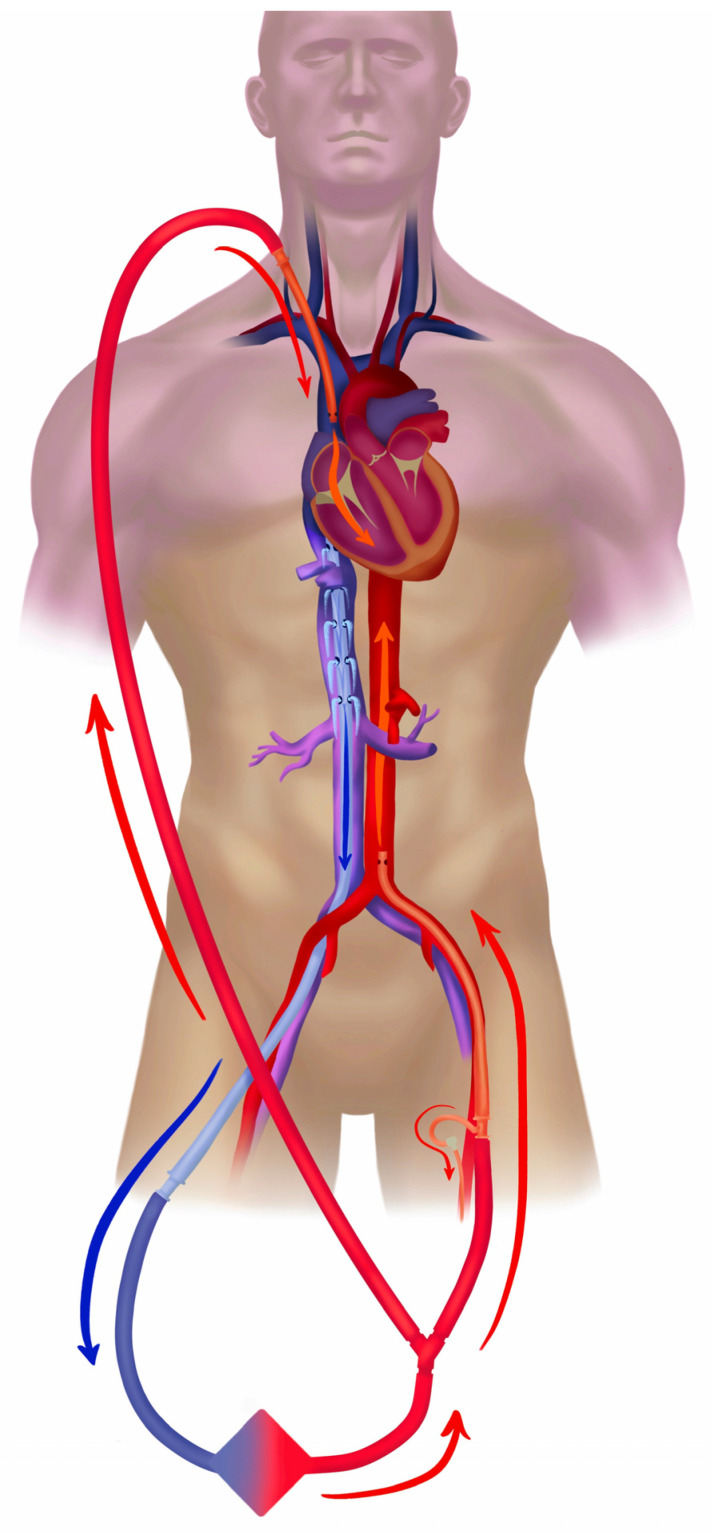
Veno-venoarterial peripheral extracorporeal membrane oxygenation. Veno-venoarterial (VVA) ECMO is a *hybrid mode*, i.e., composite of veno-venous and veno-arterial modes. In this case the drainage (blue) is from the inferior vena cava with return (red) via both the right jugular vein into the superior vena cava (offers *VV support*), and the left femoral artery (cannula tip in the iliac artery or lower abdominal aorta (*VA support*). ELSO configuration abbreviation: V_frivc_-A_fl_d_t_V_jrsvc_.

In cases of a pulmonary source of septic shock (e.g., pneumonia), the recovery of cardiac function and the restoration of pulmonary circulation are often associated with the development of differential oxygenation (“Harlequin syndrome”), and hypoxemia of the aortic arch, brain, coronary vessels, and the right arm typically ensue (Figure 1b). Differential oxygenation is concomitant with the lesser-known differential carbon dioxide tension [44], which may impact, e.g., the neuro-trauma ECMO patient, renal acid-base physiology, and breathing pattern of the spontaneously breathing patient. From the existing studies, we have strong indications that the slope of CO2 reduction after the commencement of ECMO influences the outcome [73,74]. The common treatment in femoro-femoral VA configuration is debranching of the arterial return and cannulation of the superior vena cava, typically via the right jugular vein, which, by returning 1.5–2 L/min of oxygenated blood into the pulmonary circulation, restores oxygenation in the upper body (Figure 2). This transition to the *hybrid modality* VVA, in the form of the veno-venoarterial configuration (V-VA; veno-arteriovenous V-AV), also reduces circulatory support into the aorta during the development of the hyperkinetic stage of septic shock. 

The femoral drainage cannula (depending on design) may have to be retracted to the level of the Eustachian valve in the right atrium to reduce the risk of the recirculation of the “VV” component currently applied via the hybrid mode. Moreover, introducing a Y-piece connector into the circuit for debranching increases the risk of coagulation activation, which may be further augmented by the use of a gate-clamp or Hoffman clamp to balance the venous and arterial return flow fractions (i.e., fraction of veno-venous and veno-arterial support, respectively). Even though the total ECMO flow may be unchanged, or even increased, after conversion to VVA, the pressure in the arterial return tubing may decrease and, thus, the blood flow to the cannulated leg via the distal perfusion catheter will decline. The prograde loss of flow for distal perfusion may put the cannulated leg at risk, particularly during weaning (further decreased flow) from the circulatory support (VA component) [54]. 

**Figure 3:**
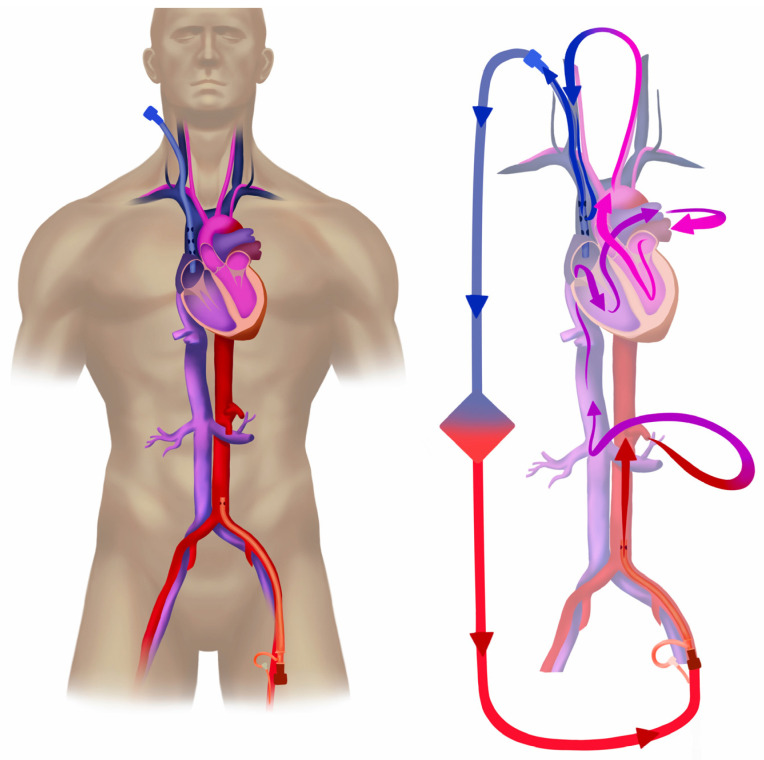
Jugulo-femoral peripheral veno-arterial extracorporeal membrane oxygenation. Arterial return is retrogradely into the iliac artery, or lower abdominal aorta. The drainage, however, is from the superior vena cava (SVC). After the hyper-oxygenated ECMO blood has perfused the lower body, the oxygen content of the blood entering the IVC on the way back to the heart is still high. With maintained cardiac output (CO) and drainage from the SVC, venous return to the heart is by blood from the IVC (high in oxygen). This blood passes through the sick lung over to the left side and subsequently perfuses the vascular areas of native CO. The venous blood returning via the SVC from these areas is then drained out to the ECMO circuit. Drainage of the most de-saturated blood provide high leverage for oxygenation over the membrane lung. ELSO configuration abbreviation: V_jra_-A_fl_d_t_.

To circumvent these risks, a simple dual site cannulation configuration is jugulo-femoral VA ECMO (Figure 3). The same can be accomplished using a long single stage (lighthouse) femoral cannula placed with the tip in the upper part of the right atrium or into the superior vena cava (SVC). A configuration with this drainage point has shown superior oxygenation of the upper body to the femoro-femoral approach with drainage from the inferior vena cava (IVC) in both animal experiments and clinical settings [54,55,56,57]. For less drainage flow resistance, a short (multi-staged or single stage) drainage cannula is placed via the right jugular vein with the tip in the upper part of the right atrium (RA). The return cannula is positioned via the femoral artery and the distal perfusion, as is the case in femoro-femoral VA ECMO. When draining the blood in the SVC, the cardiac output is maintained through the venous return of blood from the inferior vena cava. This blood is still rather high in oxygen content after the hyper-oxygenated ECMO blood has perfused the lower body (S_ivc_O_2_ 80–85%). Thus, IVC blood will enter the pulmonary circulation, and will subsequently be ejected by the left ventricle for perfusion of the upper body areas. When this blood enters the venous side (S_svc_O_2_ 60–65%), it flows towards the heart via the SVC, where it is drained out to the ECMO circuit. The capacity for net oxygen delivery through the ML is thus rather high, from a S_pre_O_2_ of 65% to a S_post_O_2_ of 100%.

**Figure 4:**
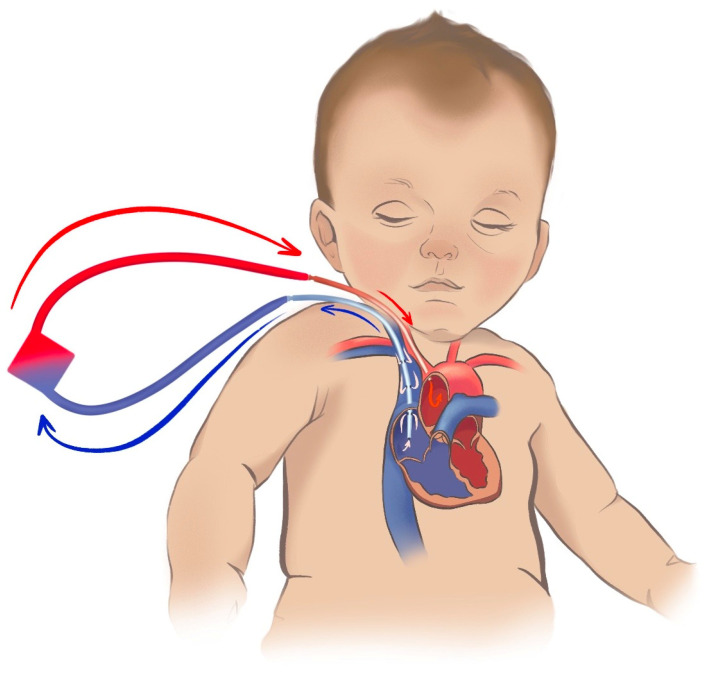
Jugulo-carotid veno-arterial extracorporeal membrane oxygenation. Peripheral atrio-carotid, or jugulo-carotid veno-arterial extracorporeal membrane oxygenation (ECMO) used in neonates and children up 15–25 kg body weight. Drainage is performed via a left jugular cannula placed with the tip in the right atrium (blue), and return (red) is via the left carotid artery with the tip inserted 5–10 mm cephalad to the aortic arch. This approach allows for mixing natively ejected blood with ECMO blood closer to the heart and reduce the area of differential oxygenation (basically the coronary arteries). A risk for cerebral thrombo-emboli via the open left carotid is present. The right carotid artery is often ligated during cannulation and could be subject to reconstruction during the explanation of ECMO. The ECMO circuit is simplified in Figure 4. ELSO configuration abbreviation: V_jra_-A_carr_.

Figure 4 shows the commonly used atrio-carotid VA configuration used in newly born and smaller children (<15–25 kg), with similar drainage point as in the jugulo-femoral approach for adults described above. In this configuration, however, the arterial return is close to the aortic arch via a blunt or single stage cannula, limiting the risk for differential oxygen and differential carbon dioxide tension. The use of this method may be restrained in children because of limitations in femoral cannulation due to the vessel size. If the return cannula has advanced too far (into the aorta), and the return flow is directed towards the aortic valve, left ventricular ballooning is a risk. Echocardiography is recommended for the monitoring of the cannula position, flow pattern, and cardiac function.

**Figure 5:**
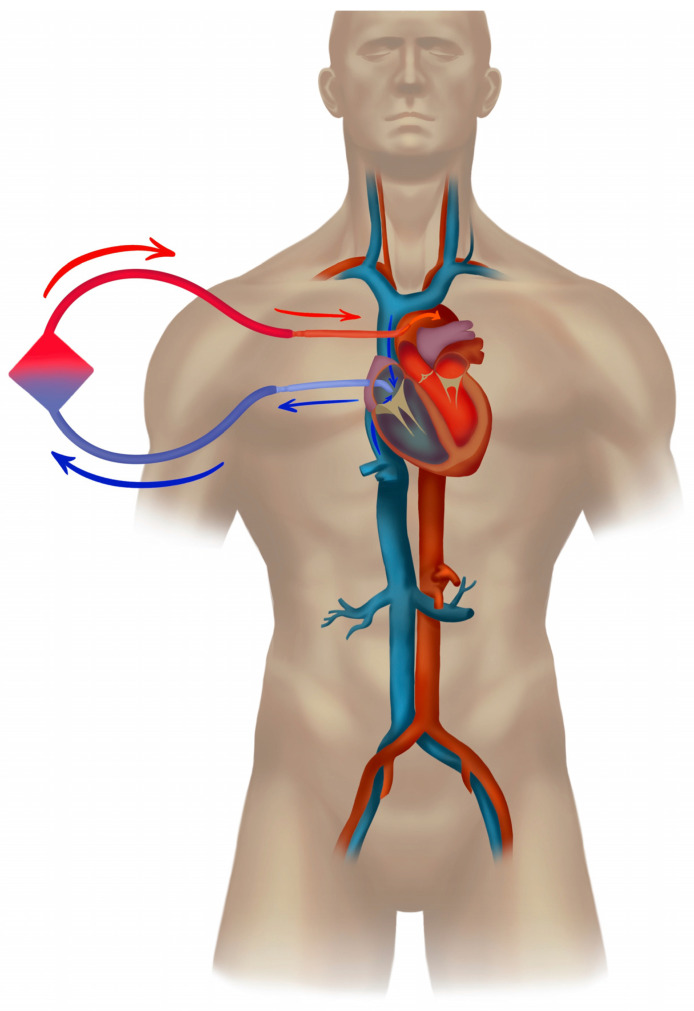
Central veno-arterial extracorporeal membrane oxygenation. Central veno-arterial (VA) extracorporeal membrane oxygenation (ECMO) enables high blood flow compared to peripheral VA ECMO. In central cannulation, blood is drained from the right atrium and returned into the ascending aorta. Sternotomy is required. In the illustration the ECMO circuit is simplified. ELSO configuration abbreviation: RA-AO.

In adult patients, however, access to larger vessels via a genuine central VA ECMO cannulation in cases of continued refractory vasoparalysis may be considered (Figure 5). However, this requires sternotomy, which is associated with further bleeding and infectious complications [75]. 

**Figure 6:**
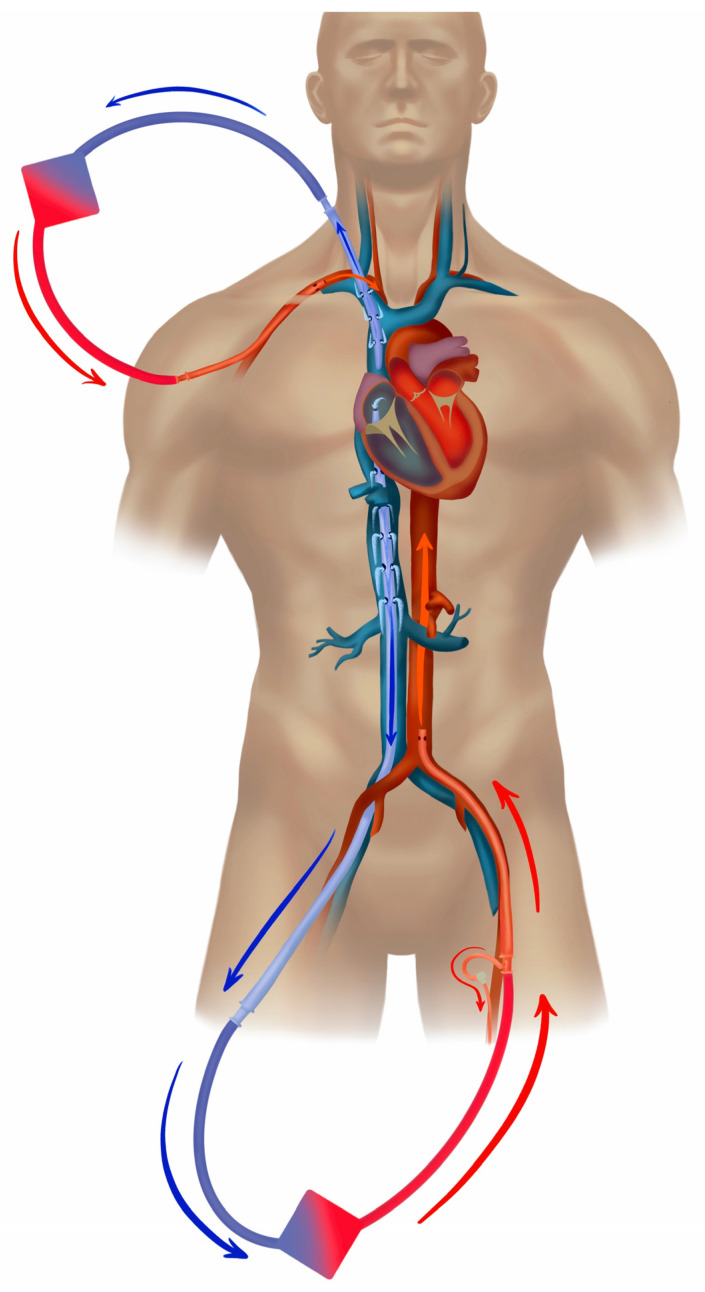
Multiple circuit extracorporeal membrane oxygenation for high oxygen delivery. Two independently working parallel veno-arterial (VA) extracorporeal membrane oxygenation (ECMO) systems (circuit + console). In addition to typical femoro-femoral VA application (lower circuit in figure), a second ECMO system is applied using jugular drainage from the superior vena cava with return to aortic arch via the right axillar or subclavian artery. This approach has been anecdotally used in hyperkinetic stage of septic shock and provides extra support to circulation and eliminates the risk of oxygen deprivation in upper body [72]. A Goretex™ graft can be applied to the side of the subclavian artery and then this *chimney graft* is cannulated. Single circuit chimney graft VA configuration is used for bridging to heart/lung transplantation as it provides easy mobilization [76]. In the illustration the ECMO circuits are simplified. ELSO configuration abbreviation: V_fr_-A_fl_d_t_\V_jr_-A_sr_.

An experimental approach with two parallel peripheral VA ECMO configurations (femoral and axillar), which allows for high ECMO blood flows and avoids sternotomy, has been successfully tested in adults (Figure 6) [72]. 

Another situation which may provide indication for VA ECMO is profound refractory vasoparalysis without cardiac compromise; those are patients with a preserved or even increased ejection fraction, a non-dilated left ventricle, maintaining normal to supranormal cardiac output. The VA ECMO circulatory support adds an extra flow into the aorta, helping to overcome vasoparalysis and to maintain the vital perfusion pressure; this may work, depending on the patient’s stress circulating blood volume, which also depends on the patient’s body mass index (BMI). Hence, children from the neonatal age up to the pediatric category (e.g., up to 35 kg body weight) may benefit from VA ECMO even in the event of hyperdynamic septic shock [33,35,77]. In adolescents and adults, the data are very limited; however, one experienced high-volume center VA ECMO in distributive shock has shown benefits, with a hospital survival of 67% [17]. Case reports and case series suggest the potential application of central VA ECMO, which requires a sternotomy, with all of the associated side effects [75].

### 2.6. Clinical Considerations

In deciding whether VA ECMO support is indicated, the heart systolic function, cardiac output, and cardiac index play critical roles. Unfortunately, no publication to date has addressed any cut off for the cardiac index, which might be judged inappropriately low to match vasoparalysis poorly responding to vasopressor therapy. The patient´s history—e.g., previous adaptation to a lower cardiac index, source of sepsis, and changes recorded with bedside echocardiography [78]—help to decide the most suitable time that the VA ECMO is indicated. 

Severe sepsis and septic shock are associated with coagulopathy, and sometimes with bone marrow suppression. Patient-tailored anticoagulation, or sometimes no anticoagulation, is indicated. A low platelet count should be weighed against the benefits of ECMO as an adverse factor for the patient´s outcome [79,80,81]. 

A loss of pulsatile flow and a non-opening aortic valve during VA ECMO support is associated with the risk of left ventricular overload and thrombus formation in the cardiac chambers [82]. Up to 10% of patients on VA ECMO may require left ventricular unloading, with additional devices like the Impella (Abiomed Inc., Danvers, MA, USA), which may be associated with side effects, especially in a pro-coagulant state such as septic shock. Nonetheless, the combination of Impella and VA ECMO (i.e., ECMELLA, or ECPELLA) is fraught with a significant incidence of hemolysis and an increased bleeding risk. The release of free hemoglobin into plasma further impairs capillary perfusion and may be linked to multiorgan failure [81]. In children, septostomy is regarded as beneficial—an intervention that does not seem to offer the same efficiency as in adults [83].

A small vessel size limits the size of the cannulas, and hence the ECMO flow, which is of critical importance to offset refractory vasoparalysis. A surge of obese and morbidly obese patients during the COVID-19 pandemic, together with the incidence of various types of stress cardiomyopathy as part of septic shock presentation, tested the limits of this approach. Typically, morbidly obese women with thin femoral vessels, and thus without an adequate blood flow matching their hyperkinetic stage of septic shock, are fraught with an increased risk of a worse outcome [84].

Moreover, most studies on ECMO and septic shock are of single-center design, and the patient numbers are limited. Due to historical development, most centers who do provide support to this patient group have been active for decades (i.e., are experienced) and would be classified as high-volume ECMO centers. This makes the generalizability of results and a recommendation for septic shock as a general indication everywhere rather limited. Advocating for large randomized controlled multi-center studies poses an ethical problem as the centers with experience seem to provide results—in terms of the ICU, hospital, and long-term survival—that are significantly higher than expected in comparison to the alternative, conventional intensive care [13,17,18,52].

The patient outcome may improve due to treatment in high-volume ECMO centers [85,86,87], where age group experience may significantly impact the outcome [88]. The data also show that the centralization of ECMO services improves resource utilization and reduces costs for society [89,90]. Mobile ECMO services have been developed for patient assessment, cannulation, and retrieval in ECMO for continued treatment in a dedicated ECMO center [86,87,88,89]. For septic shock patients, treatment should be offered in experienced high-volume centers. The patient may be transferred before ECMO commencement, but it is fully feasible to cannulate and start ECMO at the referring hospital before transport. Transport on ECMO can today be regarded as safe if performed by an experienced team [91,92,93,94]. 

The potential benefits and drawbacks of VA ECMO in septic shock are summarized in Table 2. 

### 2.7. Anticoagulation Management

Anticoagulation during ECMO in septic shock follows the protocol, where each patient has a customized dose decided at daily contextual roundings. The frequency of monitoring using laboratory assays may be as close as hourly depending on the situation. There is no *gold standard* for anticoagulation or methods of monitoring. The most commonly used drug for anticoagulation, both in children and adults, is still unfractionated heparin (UFH), which acts via the potentiation of antithrombin (AT). Direct thrombin inhibitors (DTIs), such as bivalirudin and argatroban, are AT independent and their use is increasing. The action of DTI is on thrombin only, and the monitoring is similar to that of UFH, i.e., through the activated partial thromboplastin time. Other feasible monitoring modalities during UFH-anticoagulation include the assessment of activated Factor Xa and viscoelastic methods. The ELSO Anticoagulation Guideline for adult and pediatric ECMO was revised and published online in 2021 (file://gainaskar01.gaia.sll.se/fs_kar_usr$/511p/Download/2021_elso_adult_and_pediatric_anticoagulation.1%20(1).pdf (accessed on 7 October 2023)).

### 2.8. Complications

The number of works focusing on or reporting complications in VA ECMO for septic shock is low. The pathophysiology for cardiac failure in sepsis is also different from that of ischemic heart disease. Thus, complications such as the need for the unloading of the left ventricle or pulmonary edema is rarely heard of [18,95]. Adult studies on septic shock report ischemia of the cannulated leg in about 5% of all cases, cannulation site bleeding in 5–21% of cases, and a risk for cerebral complications (ischemic lesions, bleedings) in 8–20% [17,18,52,95]. Infections of different definitions are reported in 8–21% of cases (ventilator induced pneumonia, cannula site infection, sepsis). In the majority of patients where treatment was withdrawn, the cause of futility was intra-cranial bleeding or an ischemic stroke [17].

Concerning the placement of the return cannula via the carotid artery in newly born and smaller children, studies based on the ELSO Registry are ambiguous in terms of the increased risk of stroke [96,97]. In a recent work on brain infarctions in neonatal ECMO, the sepsis-related severity of illness, presence of VA ECMO, conversion of the ECMO modality, CRRT, and extracranial thrombosis were independent risk factors for a cerebral event [98]. In septic children, Melnikov et al. reported cannula-related clotting problems in 13%, but no association between complications and death [13]. In the review and meta-analysis by Ramanathan et al. [45], 14 studies (246 patients) reported a complication rate of 31%; 40% percent were hemorrhagic, 21% neurologic, 12% vascular, 5% renal, and 1% infectious. The summarized mechanical and circuit complication rate (clotting, oxygenation failures, pump and heat exchanger malfunctions, air in circuits) was 46%.

## 3. Conclusions

In severe septic shock with vasoparalysis requiring vasopressors equivalent to a vasopressor score of >30-50, the echocardiographic assessment of the hemodynamics should be obtained intermittently and used for the calibration of a continuous method for cardiac output monitoring. If the cardiac index approaches subnormal levels (CI < 2.5 L/min m^−2^), an expert opinion should be sought to consider an indication for VA ECMO support. Further progress towards cardiogenic shock (CI < 1.8 L/min m^−2^) in septic shock, regardless of inotropic support (e.g., levosimendan, dobutamine), should be regarded as an indication for VA ECMO support. The use of ECMO in hypodynamic septic shock has shown benefits in avoiding circulatory collapse and cardiac arrest with a high survival rate. In patients who maintain a high cardiac index as part of refractory vasoparalysis, VA ECMO continues to be reserved for children and patients with a lower body mass index, and its use is reserved for experienced centers. 

## Figures and Tables

**Table 1:** Respiratory and circulatory indicators for extracorporeal support in refractoriness.

Age Group	Respiratory	Cardiac
Neonates (0–28 days)	Inadequate tissue oxygen delivery despite maximal therapy (increasing lactate, metabolic acidosis, signs of end-organ dysfunction); Severe hypoxic respiratory failure (p_a_O_2_ < 40 mmHg); Oxygenation Index (OI) > 40 with no improvement; Severe pulmonary hypertension with evidence of RV a/o LV dysfunction. OI = [Mean airway pressure (cmH_2_O) × FiO_2_(%)]/paO_2_ (mmHg)	Persistent systemic systolic pressure < 50 mmHg, or age dependent MAP (mmHg) equivalent to GA in weeks) despite adequate fluid resuscitation; use of vasopressors; urine output < 1 mL/kg h^−1^; altered mental status (due to low cardiac output)
Pediatric (1 mth-18 years)	Acute severe respiratory failure with progression despite optimized conventional therapy; ECMO consideration if mortality risk >50%, and strongly recommended if mortality risk approaches 80% with conventional therapy; Earlier consideration to minimize barotrauma and other morbidities due to aggressive conventional therapies.	Persistent systemic systolic pressure <50 mmHg despite adequate fluid resuscitation; use of vasopressors; urine output < 1 mL/kg h^−1^; altered mental status (due to low cardiac output)
Adult (>18 years)	Hypoxemic respiratory failure (P_a_O_2_/FiO_2_ < 80 mmHg) despite optimum conventional treatment including trial of prone positioning; Hypercapnic respiratory failure (pH < 7.10–7.25) despite optimal conventional mechanical ventilation	Systemic systolic pressure < 90 mmHg or MAP < 70 mmHg despite adequate fluid resuscitation; Vasoactive inotropic score [44] >50, 1 h; >45, 8 h; urine output < 30 mL/h; lactate > 2 mmol/L; Cardiac Index < 1.8–2.1 L/min m^−2^; S_v_O_2_ < 55%; altered mental status

Abbreviations: ECMO, extracorporeal membrane oxygenation; GA, gestational age; MAP, mean arterial blood pressure.

**Table 2:** Potential benefits and drawbacks of VA ECMO in septic shock.

VA ECMO in Severe Septic Shock	Potential Benefits	Potential Side Effects
Circulation with low CI (<2.5 L/min m^−2^)	Substitution of CI, maintenance of perfusion pressure, decatecholaminization	Increased LV afterload
Circulation with normal to high CI	Maintenance of perfusion pressure and peripheral perfusion, decatecholaminization	Increased LV afterload, benefits limited by high BMI and requirements for a high circulating stress blood volume
Ventilation	Enables lung protective ventilation	Lung hypoperfusion, V/Q mismatch
Oxygenation, systemic DO_2_	Restoration of peripheral DO_2_ depending on circulation	Differential oxygenation (Harlequin syndrome) requiring VVA and weaning via VV ECMO
Coagulation	-	Risk of bleeding, especially in septic bone marrow suppression, thrombocytopenia, and coagulopathy
Hemolysis	-	High pfHb, especially on ECPELLA

**Abbreviations:** BMI, body mass index; CI, cardiac index; DO_2_, distributed amount of oxygen; ECMO, extracorporeal membrane oxygenation; ECPELLA, combination of Impella (Abiomed Inc., Danvers, MA, USA) and VA ECMO; pfHb, plasma free hemoglobin; LV, left cardiac ventricle; VA, veno-arterial; V/Q, ventilation perfusion quotient; VV, veno-venous.

## Data Availability

Not applicable.

## Supplementary Materials

The following supporting information can be downloaded at: https://www.mdpi.com/article/10.3390/jcm12206661/s1, Table S1: Included articles with clinically related inclusion and outcome data; Table S2: Cannulation strategies.
