# Supplementary material for: Extracorporeal Membrane Oxygenation for Septic Shock in Adults and Children: A Narrative Review
Source: J Clin Med. 2023 Oct 20;12(20):6661. doi: 10.3390/jcm12206661 (PMC10607553; doi:10.3390/jcm12206661)
Supplement: Supplementary file 1 [file jcm-12-06661-s001.zip › Table S1.pdf]

Table S1. Table shows included articles with clinically related inclusion and outcome data

Author	Age group	N	Mode	LVEF	SOFA	Weaned alive	Survival to discharge	Associations with death	Comment
Bréchot, et al. 2013 [18]	Adult	14	VA	16%	18	84%	71%	n/a	Femoro-femoral cannulation; SAPS-3: 84
Bréchot, et al. 2020 [95]	Adult	82 (5-center)	VA (VVA n=8)	17%	17	74%	60%	n/a	Femoro-femoral, 13% CA before cannulation.
Falk, et al. 2019 [17]	Adult	20	VA (n=18), VV (n=2)	25%	16	90% (VV 50%; VA 94%)	75%	Need of VV to VA conversion; Number of organ failures	Jugulo-femoral VA, jugulo-femoral VV; 7 pt CA before ECMO, survival 71%; SAPS-3: 86 1 VV converted to VA
		17	VA (n=9), VV (n=8)	52%		65% (VV 62%, VA 67%)	65%		Jugulo-femoral VA, jugulo-femoral VV; 5 VV patients converted to VA.
Huang, et al. 2013 [26]	Adult	52	VA	>55%	17	n/a	15%	Age (all >60 years died n=20);	Peripheral femoro-femoral cannulation; 40% CA before ECMO start, 10% survival; no need of LV venting
Park, et al. 2015 [27]	Adult	32	VA	25%	16	41%	22%*	Lactate, SOFA, troponinT, CA (HR 4.6), time to ECMO	Femoro-femoral cannulation; 44% CA before ECMO start; SAPS-3: 79
Vogel, et al. 2018 [52]	Adult	12	VVA	16%	10	75%	75%	n/a	Femoro-femorofemoral cannulation; 42% CA before start; 1 pt neded IABP for LV unloading
MacLaren, et al (2007) [14]	Ped	45	VA (central n=11, periphleral n=34)	n/a	MAP 45 mmHg PaO2/FiO2 ratio 64 mmHg; Lactate 8.1 mmol/L	n/a	47%; (73% central, 44% peripheral)	Benefit for central cannulation	Jugulo-carotic, femoro-carotic, right atrium to aorta.

MacLaren, et al (2011) [15]	Ped	23	VA (central)	n/a	MAP 57 mmHg PaO2/FiO2 ratio 99 mmHg; Lactate 7.5 mmol/L	78%	74%	High lactate before	35% CA before ECMO
Melnikov, et al. 2022 [13]	Ped	31	VA, VV	n/a	MAP 51 mmHg PaO2/FiO2 ratio 48 mmHg PIM2/3, 31.6 EMR% Lactate 8.5 mmol/L	71%	68% (VA 62%, VV 80%)	High lactate and creatinine before	Jugulo-femoral VA; juguloa-femoral VV; 29% CA before cannulation.
Ramanathan, et al. (2020) [45]	Ped/ Neo	2559	n/a	n/a	n/a	Overall 59%; VA 65% (n=208); Ped 50% (n=138); Neonates 73% (n=85)	n/a	No variable predictive of mortality	Review and meta-analysis
Skinner, et al. (2012) [16]	Ped/ Neo	4332	VA (n=3256), VV (n=1076)	non-cardiac septic shock	n/a	Overall 68% (VA 64%, VV 79%); Ped 1 mth-12 yrs VV 54% (n=63); VA 37% (n=169); Ped >12 yrs VV 29% (n=9); VA 43% (n=25;) Neonates VV 83% (n=777); VA 70% (n=1892)	n/a	VA (odds ratio, 2.06; 95% CI, 1.74-2.44; p<0.001	ELSO Registry study

Abbrevitions: CA, cardiac arrest; CI, confidence interval; ECMO, extracorporeal membrane oxygenation; ELSO, Extracorporela Life Support Organization; EMR%, estimated mortality rate (in percent); LV, left cardiac ventricle; LVEF, left ventricular ejection fraction; MAP, mean arterialblood pressure; N, total number; Neo, neonate; Ped, pediatric; PIM, Pediatric Index of Mortality; SAPS-3, Simplified Acute Physiology Score; SOFA, Sequential Organ Failure Assessment; VA, veno-arterial; VV, veno-venous; VVA, veno-venoarterial
