# Supplementary material for: Extracorporeal Membrane Oxygenation for Septic Shock in Adults and Children: A Narrative Review
Source: J Clin Med. 2023 Oct 20;12(20):6661. doi: 10.3390/jcm12206661 (PMC10607553; doi:10.3390/jcm12206661)
Supplement: Supplementary file 1 [file jcm-12-06661-s001.zip › Table S2.pdf]

Table S2. Cannulation strategies

Age group	Mode	Configuration	Cannula size and design	Cannula tip position	Comments, benefits and drawbacks
**Neonate (0-28 days)**	VA	Jugulo-carotid, (V_j-A_{car})	D: 8-14Fr (SS, 1/4" tubing); R: 6-12 Fr (SS, 1/4")	D: inside RA; R: via right carotid artery ca 5-10 mm cephalad to aortic arch	Malpositioning of return cannula (too deep) may increase afterload significantly depending on return jet direction. Echocardiography for monitoring is adviced.
**Pediatric (1 mth-18 y)**	VA	Jugulo-carotid VA (<15-25 kg), (V_j-A_{car})	D: 8-15Fr (SS, 1/4"), 15-19Fr (SS, 3/8" tubing); R: 8-15Fr (SS, 1/4"), 15-17Fr (SS, 3/8")	D: inside RA. R: via right carotid artery advnced no further than 5-10 mm cephalad to aortic arch	For improved drainage flow, 3/8" tubing may be used with ≥15Fr draiange cannula whereas return may be kept using 1/4" tubing and 14Fr return cannula in the lower age/weight range. May require down-sizing from 3/8" to 1/4" before the membrane lung.
**For children >15-25 kg additional modes and configurations, see *Adult* below**					
**Adult (>18 y)**	VA	Jugulo-femoral, (V_j-A_f)	D: 15-25Fr (15-23cm, SS), 19-29Fr (50-55cm, SS), 21-25Fr (38cm, MS); R: 15-19(21)Fr (18-23cm, SS)	D: Cannula tip inside upper part RA. The MS cannula will have higher fractional driange of SVC blood and less of the RA mix compared to SS cannula placed in RA. R: iliac artery	(1) Drainage zone in the IVC (deliberate, or accidental by cannula or diaphragmatic migration), increases risk of development of severe form of Differential oxygenation. Differential oxygenation will always be more pronounced in ICV drainage compared to SVC drainage in femoral return peripheral VA ECMO [54]. (2) Consider distal perfusion catheter for cannulated leg. (3) If a larger size femoral return cannula is used, besides risk of Femoral artery obstruction, the lower pressure drop over the larger cannula may limit the distal perfusion catheter flow and pose a risk for the cannulated leg. Flow in this catheter should be measured continuously.
		Femoro-femoral (Vf-Af)	D: 19-29Fr (50-55cm, SS); 21-29Fr (38-61cm, MS); R: 15-19(21)Fr (18-25cm, SS)	D: tip in upper part of IVC, better in upper part of RA or pass into/above junction of SVC/RA. R: iliac artery	
		Venoveno-arterial, i.e. dual-drainage/bi-caval drainage VA mode, (V_jV_f-A_f)	Cannula size *(found directly above)* is determined by vessel size and estimated need of flow.	A singlestage cannula will drain close to the tip. A multistage cannula will drain the most from the most proximal open sideholes, and at increasing flow rates the mean drainage point will move closer to the tip [59].	(1) Both the use of two single-lumen drainage cannulae or one bi-caval drainage cannula, fractional SVC/IVC flow is dependent on total ECMO flow which may obscure interpretation of Differential oxygenation over the flow range [54,61]. (2) Bi-caval multistaged interrupted cannulae may not be approved for use >6h, i.e. CBP. (3) Distal perfusion catheter for cannulated leg, see *VA ECMO* above.
	Hybrid mode: VVA	Veno-venoarterial, (V-VA, V-AV)	Composite of VV and VA ECMO, thus one common drainage and dual return according to VA and VV above.		(1) Return flow is devided to both arterial and venous side which requires balancing of "A" and "V" flow, respectively, due to different compartmental blood pressures. This may be accomplished using either Gateclamp or Hoffman clamp (turbulence at this site increases risk of platelet activation, and hemolysis). (2) Distal perfusion of cannulated leg may be impaired due to decreased return cannula pressure gradient.

Table S2. Knowledge of cannula design and positioning is crucial to understand what the offered ECMO support will and can accomplish concerning oxygen delivery and oxygen distribution for the patient. The drainage cannula is the most important cannula not only limiting drainage, i.e. ECMO blood flow, but also overall oxygen distribution in the patient [54-58]. Aim to apply the largest drainage (venous) cannula possible for optimum drainage, i.e. low applied suction pressure (≥ -80 mmHg). In the decision-making process, ultrasound/echocardiography is an important tool to asses cardiac function and vessel size(s).

Abbreviations: CBP, cardiopulmonary by-pass; D, drainage; Fr, French, 1 Fr = 1/3 mm in cannula outer diameter; IVC, inferior vena cava; MS, multistage cannual; R, return; RA, right atrium; SS, single stage cannula; SVC, superior vena cava; VA, veno-arterial; VVA, veno-venoarterial
